# Mother surrogacy in Albania: a one-way ticket

**DOI:** 10.3389/fgwh.2025.1570513

**Published:** 2025-06-05

**Authors:** Gentian Vyshka, Ermir Roçi, Entela Basha

**Affiliations:** ^1^ Biomedical and Experimental Department, University of Medicine, Tirana, Albania; ^2^ Division of Neurosciences, University Hospital Center “Mother Teresa”, Tirana, Albania

**Keywords:** mother surrogacy, Albania, regulatory means, ethical issues, motherhood

## Introduction

The problem of legalizing or promulgating regulatory means for mother surrogacy in Albania has recently attracted public attention. For a country with large social problems, jumping into discussions on intricate technology and ethics seemed unlikely, and the issue was immediately polarized.

The first negative responders to the idea of adopting legislative measures in favor of mother surrogacy were religious actors (1–3). Almost simultaneously, representatives of Christian and Muslim communities (the latter being the major religion in the country) positioned themselves against the idea based on religious rulings.

Gaps into the local legislation in Albania are palpable and of major concerns, since the matter is not regulated by law (4). The Family Code of Albania mentions the term in its article 261 but by no way provides any regulatory means, neither for commercial nor for altruistic surrogacy (4, 5). If not permitted, and not illegal at the same time, a gray zone is denoted from sources that reasonably have already formulated troublesome deviations that might produce such a situation (6).

While trying to provide our perspective, we focused on terminology, language, and everyday speech. In fact, the public has already reacted with aversion to a very specific dilemma, such as this of surrogacy, without making any distinction between the commercial (the so-called *paid surrogacy*) or the altruistic form (the *un-paid surrogacy*, when the mother receives no financial compensation) of the practice.

### In the beginning was the Word^1^

The way something is called, especially in everyday life and lay language or vocabulary, speaks a lot about how people conceive and perceive the whole of it. Albanian has a striking similarity between words denoting adoption and adaptation (respectively: *adoptim* and *adaptim*), while their respective meanings differ substantially. To address the problem, the legislator has largely adopted the term *birësim* (equivalent to child adoption), originating from *bir* (the Latin equivalent of *filius*, according to some sources) (7).

The Table 1, in a simplified form (see “Table 1”), includes some terms related to the issue of concern. Due to the diversity of reasons, everyday Albanian has created confusion within terms. This might be an overrated presumption, especially when dealing with words borrowed from neighboring languages. What for Romanians is a term denoting a child (*copil, copilaș*), in Albanian and in other unrelated Slavic languages, is equivalent to an illegitimate offspring. It might be difficult to believe that there is such a deviation from the original meaning, but this is not the only reason for the linguistic confusion.

**Table 1:** Brief summary of terms of interest (7, 8).

Albanian term	English translation
*Surrogat*	Surrogate
*Adoptim*	Adoption
*Adaptim*	Adaptation
*Birësim*	Child adoption

Starting with the main theme, we checked the published dictionaries in Albanian from official sources (the National Academy of Sciences). The term *surrogat* (Albanian for surrogate) was not included in the printed dictionary of 1954; the definitions were almost identical in the printed editions of 1980, 1984, and 2006 (with no later editions available, to the best of our knowledge) (Box 1).

BOX 1 **Explanation of the term “surrogat” in Albanian.**

"SURROGAT,∼I m.sh. ∼E, ET.

1. Ushqim ose prodhim tjetër që zëvendëson diçka natyrore, por që nuk i ka të gjitha vetitë e vlerat e saj; mospërf. mall i lirë e i dobët. Surrogat i kafes. Surrogat gjalpi.

2. fig. Diçka pa vlerë që vetëm ngjason me diçka tjetër të vërtetë. Surrogate të kulturës"?

The snapshot from the Dictionary of the Albanian Language of 1984 grants an explanation almost identical to the recently mentioned printed dictionary (2006) and online sources (appearing later). What follows is a verbatim translation of the original definitions into English from the 1984 and 2006 editions (8):

**SURROGAT,-I** *m (masculine) (***-E***,* **-ET** *in plural)*

1. A food or other product replacing something natural, but that has not all qualities and values of the same; *pejoratively*: a merchandise of low price and not qualitative. *Coffee surrogate. Butter surrogate*.
2. *Figuratively*: Something without intrinsic values, that only resembles an original one. *Cultural surrogates*.

As such, the meaning and everyday use of the term *surrogate* in everyday Albanian is the first big obstacle toward accepting the unacceptable from a social point of view.

The everyday reality nevertheless seems different, and hardly reflects the societal reluctance or hesitancies. What is running below the ground seems to be a very profitable industry of *in vitro* fertilization technologies, acting behind closed doors and taking advantage of serious legislative lacunae: a recent TV program on a nationwide channel thoroughly considered the issue (9).

### Toward a catastrophic form of mothering?

In another small step, one might challenge the adversaries of the mother's surrogacy as being contrary to technological advancements. Nothing new or unique to the country. However, silencing these voices would not only require waving the flag of a big technological step, leading toward the blue horizon. The reality is, in fact, full of ethical, legal, and–if you want–even uncertainties from a technical perspective.

Legal controversies are much more acute and sharp than ethical controversies; hence, the strict positions of some countries clearly abolish the surrogate option of mothering. Religious backgrounds do not differ substantially among the three monotheistic faiths prevalent in Albania, rendering this option incompatible with the processing of faith. Relating the Divine to the mother and mothering has a very long ecclesiastic tradition (10). In canonical Islamic texts, mothers are described as objects of veneration (11).

Thus, cultural and religious traditions leave little space, if any, for *upending* a millenarian tradition—the equivalent to *catastrophizing* in Greek language.

A reckless position appears to have been adopted when considering surrogate mothers *per se*. The majority of discussions have focused on the impact on society and family in general. How will societal concerns be addressed, and how will this affect future generations? Are we drafting a substantial change in the family structure if not already done? The potential (future) surrogate mother must have been at the center of all discussions and care. The psychological impact of surrendering progeny owing to contractual obligations has notorious predecessors. Maybe any reference to the “*Stolen babies*” is haunting: the Franco era is a very recent testimony on how newborn babies started being separated from their parents at birth (12). The history is rife with child-taking misdeeds, sometimes upgraded as a strange and unique doctrine, even under clerical disguise. *Finaly affair* could be an isolated phenomenon, if there were not an abundance of similar occurrences (13, 14).

Considered as a “world capital for surrogacy”, India has legalized right from 2002 the commercial form of surrogacy (15, 16). For a huge country like India, it seemed quite soon that legislative changes were unavoidable: foreign clients were excluded in 2015 and the concept of altruistic surrogacy was later included (17). While sources do acknowledge the widespread availability of surrogacy in India (technical and human resources, economic advantages and societal acceptance), authors converge at the necessity of avoiding exploitation of women. Hence, generally the contradicted concept remains within the frame of women's right and morality, somehow distant from medical, ethical and bioethical suspicions.

Of course, drawing comparisons between a completely different reality such as the Indian one, and European countries (here including Albania), might be defective. Extensive reviews and analyses of the policies are available, and while almost all European countries have banned commercial surrogacy, with few of them permitting the altruistic form only (Netherlands, Denmark, Czech Republic, UK), some exceptions are worth of mentioning. Russia, Ukraine are permissive to both forms (commercial and altruistic) of surrogacy, with authors raising a red flag for Ukraine as “becoming a commercial surrogacy “promised land”, now that other countries have banned or severely restricted the practice” (18).

### Regional setting: the present and the past

Italy has strictly prohibited this procedure; furthermore, it has taken regulatory steps to deter couples from undertaking surrogacy abroad (19). The Croatian Family Act explicitly prohibits surrogacy arrangements, and authors have made extensive suggestions for regulating the issue (20). Worth mentioning, Croatia has enacted an *ad hoc* law on medically assisted fertilization on 2012 (21). Greece has made substantial reforms to the Family Law Book of the Civil Code, put into effect in 1946; however, all these attempts are still subject to criticism (22).

Evidently, the landscape is not uniform, even in countries neighboring Albania. Thus, given the regional discrepancies and contradictory ways in which others address the same issue, tourist surrogacy could flourish. Owing to large regulatory lacunae and leaks, it is difficult to believe that beneficiaries and surrogate mothers will sleep in a bed of roses.

The Albanian tradition is rich with epics of a suffered motherhood (due to interpersonal issues, rather than to a medical condition), with a very particular occurrence of having no possibility to take care of or even contacting the progeny. The legend of Rozafa, a young mother who was sacrificed and buried in the walls of the castle so that it would remain strong, has clear historical implications (23). She accepted the sacrifice under one condition: her husband and his two brothers (i.e., her brothers-in-law who, in fact, orchestrated the plot) would leave a hole for her right breast so her newborn son could feed, another hole for her right hand to caress him, and a third hole for her right foot to move his cradle. The castle never collapsed (23).

It is likely that within the tribal family, there was a surreptitious war and a subconscious desire to kidnap the child of the future-to-be sacrificed and half-buried female victim. The oldest of her sisters-in-law was barren, whereas the other pretended (it seems) to be pregnant. Of course, the legend is much more complicated and multifaceted, as with all other fictional playgrounds of the epos.

The half-buried mother might seem as an opaque predecessor to forced surrogacy: You give birth to the child. You feed it. However, you do not possess it. A Freudian approach will serve as context to a highly debatable episode: do not forget that human sacrifices were rare, if non-existent, in the Hellenic epos (24). Thus, for a young mother to be buried in a legendary sacrifice for a castle under construction, while someone planned to kidnap her child, one might suppose another mechanism of intrigue and crime.

## Conclusions

There are clear reasons why legislative bodies in Albania actually tried to intervene on this issue. The decline in the natural birth rate and increased population aging has not spared our country (25, 26). Forty years after the first worldwide gestational surrogacy was reported, the country might have reached an age for such challenges (27).

In addition, legal dilemmas have been booming ever since (28). Law professionals have already decried hazardous prospective actions. In an exhaustive review published in the Albanian journal of The Chamber of Advocacy, authors conclusively emphasize that the status of a child cannot undergo a simple process of negotiation between interested parties (29).

The issue being highly controversial, positions and attempts to abolish completely surrogacy reached their peak with the Declaration of Casablanca in 2023. Olivia Maurel, the spokesperson of the same while further elaborating the statement, declared:

Albania, are you willing to open your country to the relentless surrogacy market that will put Albanian women at huge risk of exploitation and trafficking? I hope the answer to this question is no (30).

A clear-cut legal positioning would avoid all confusion and impede attempts—if any—to perform surrogacy in a cover form in Albania. If we refer to countries very close to us, just recently (October 2024) the Italian Senate has promulgated a bill considering surrogacy “a *universal crime*”, thus granting extraterritorial scope to penal prosecution (31). Drastic as it might be and ethically questionable, it seems by far the only unequivocal solution closing the door to any mischievous forms of deceptions.

Obviously, we cannot underestimate the economic effects of surrogate parenthood and the enticement its commodification brings, whose pressure could overcome the ethical dilemmas of hiring one's womb. This becomes more acute for Albania, especially when neighboring countries have adopted serious restrictions, or complete prohibition of the procedure. If instead, a small country would chose to become a sanctuary for potential surrogate mothers, this might be too much of a dream, or of a nightmare, if you wish.

## Footnotes

^1^ https://biblehub.com/john/1-1.htm
